# The impact of transmission mode on the evolution of benefits provided by microbial symbionts

**DOI:** 10.1002/ece3.1166

**Published:** 2014-08-06

**Authors:** Jason W Shapiro, Paul E Turner

**Affiliations:** 1 Department of Ecology & Evolutionary Biology, Yale UniversityNew Haven, Connecticut

**Keywords:** By-products, mutualism, parasitism, partner choice, pleiotropy, symbiosis

## Abstract

While past work has often examined the effects of transmission mode on virulence evolution in parasites, few studies have explored the impact of horizontal transmission on the evolution of benefits conferred by a symbiont to its host. Here, we identify three mechanisms that create a positive covariance between horizontal transmission and symbiont-provided benefits: pleiotropy within the symbiont genome, partner choice by the host, and consumption of host waste by-products by symbionts. We modify a susceptible-infected model to incorporate the details of each mechanism and examine the evolution of symbiont benefits given variation in either the immigration rate of susceptible hosts or the rate of successful vertical transmission. We find conditions for each case under which greater opportunity for horizontal transmission (higher migration rate) favors the evolution of mutualism. Further, we find the surprising result that vertical transmission can inhibit the evolution of benefits provided by symbionts to hosts when horizontal transmission and symbiont-provided benefits are positively correlated. These predictions may apply to a number of natural systems, and the results may explain why many mutualisms that rely on partner choice often lack a mechanism for vertical transmission.

## Introduction

Symbioses – interactions in which two species live together for at least part of their life cycles – can range from obligate parasitism (e.g., Ebola virus infecting humans) to obligate mutualism (e.g., *Buchnera aphidicola* bacteria in pea aphids, Moran et al. 2008). Abundant prior research has examined the role of transmission mode in symbiont evolution, although the focus has often been on potential trade-offs between horizontal transmission and virulence evolution in parasites (May and Anderson 1983; Ewald 1987; Bull et al. 1991; Bull 1994; Ebert 1994; Read 1994; Lipsitch et al. 1995, 1996; Agnew and Koella 1997; Turner et al. 1998; Messenger et al. 1999; Day 2001; Ferdy and Godelle 2005; Stewart et al. 2005; Smith 2007; Alizon et al. 2009). Further, it is often expected that vertical transmission will favor the evolution and persistence of mutualistic symbioses (Ewald 1987; Doebeli and Knowlton 1998; Herre et al. 1999; Wilkinson and Sherratt 2001; Foster and Wenseleers 2006; Sachs et al. 2011b). Yet, many mutualistic symbionts are regularly transmitted horizontally (Genkai-Kato and Yamamura 1999; Wilkinson and Sherratt 2001; Kikuchi et al. 2007; Usher et al. 2007; Sachs et al. 2011a), and some lack vertical transmission entirely (e.g. Kikuchi et al. 2007). Examples of horizontally transmitted symbionts include mycorrhizal fungi (Johnson et al. 1997), gut microbiota (Backhed et al. 2005), coral symbionts (Day et al. 2008), *Vibrio fischeri* in squids (Ruby 1996), *Hamiltonella defensa* in aphids (Vorburger and Gouskov 2011), and conjugative plasmids (Turner et al. 1998; Dionisio et al. 2005). How horizontal transmission affects the evolution of symbiont-provided benefits has remained an open question.

The evolution of stable mutualism is challenging to explain, as all mutualisms are potentially susceptible to invasion by unhelpful cheaters. Several mechanisms have been proposed to stabilize mutualisms: partner choice, where one or both species favor interactions with more helpful partners (often called sanctions if this is caused by punishing less helpful partners); by-products, where one or both species utilize waste by-products produced by the other species; and partner fidelity feedback, where interactions between partners are correlated over time via mechanisms such as vertical transmission (Foster and Wenseleers 2006; Sachs et al. 2011b).

Horizontally transmitted mutualisms are especially puzzling, because they appear to disrupt the partner fidelity feedbacks generally thought to stabilize mutualisms. Further, the common assumption that increased horizontal transmission selects for costlier symbionts (i.e., the virulence-transmission trade-off hypothesis) hints that horizontally transmitted mutualists may be unlikely to persist. Some past work has suggested that horizontal transmission may mimic “pseudo-vertical transmission” (Wilkinson and Sherratt 2001) or reinforce selection imposed by vertical transmission (Lipsitch et al. 1996). Here, we examine whether horizontal transmission itself may enhance the evolution of mutualism.

In the virulence literature, mechanisms connecting horizontal transmission to interaction costs are easy to propose and are largely based on the idea that greater parasite reproduction (which enhances horizontal transmission) entails greater host exploitation. Analogous mechanisms that create a positive covariance between horizontal transmission and symbiont-provided benefits, however, have been underexplored. We consider three scenarios in which horizontal transmission and benefits provided by symbionts should be positively correlated. First, it is possible for pleiotropy to create a positive covariance between infectivity and symbiont-provided benefits. For instance, in the bobtail squid, *Euprymna scolopes*, which acquires its luminescent symbiont, *V. fischeri*, from the environment, both symbiont luminescence and symbiont motility are correlated with successful colonization of the host (Visick et al. 2000; Foster et al. 2004; Millikan and Ruby 2004). And, in the conjugative plasmid pB15 of *Escherichia coli*, tetracycline resistance and conjugation rate are positively correlated (Turner et al. 1998, in press[Bibr b47]). Second, partner choice also creates a positive covariance between transmission success and benefits when hosts favor infection by more beneficial symbionts. In *Acromyrmex* species of leaf-cutter ants, ants may preferentially cultivate fungus harboring more beneficial strains of bacteria, directly tying bacterial transmission to their benefits (Zhang et al. 2007). Moreover, in the aforementioned *V. fischeri* example, reduced squid colonization by less luminescent symbiotic bacteria may also reflect host rejection of deficient symbionts (Visick and McFall-Ngai 2000; Visick et al. 2000). Third, when symbionts utilize toxic host by-products, greater exploitation of the host may result in both increased symbiont fecundity (enhancing horizontal transmission) and in greater host fitness (by removing the toxic waste). This effect creates a positive feedback between partner fitness without relying on vertical transmission. This form of by-product mutualism may be particularly important when symbionts aid in digestion, as in the case of bacteria within the marine oligochaete, *Olavius algarvensis*, which lacks an excretory system (Woyke et al. 2006).

Each of these three mechanisms creates a positive correlation between symbiont horizontal transmission success and the benefits the symbiont provides to its host, but little research has investigated how these benefits should evolve in response to variation in susceptible host availability. Here, we explore the impact of variation in the rates of horizontal and vertical transmission on the evolution of benefits provided by symbionts to hosts in the context of the three mechanisms introduced above. We begin with a general susceptible-infected (SI) model and incorporate, in turn, the key features particular to the biology of pleiotropy, partner choice, and by-product interactions. We then use an adaptive dynamics approach to examine the evolution of symbiont-provided benefits as a function of both susceptible host immigration and the rate of vertical transmission. In general, we find that increased opportunities for horizontal transmission can promote the evolution of mutualism, even when also increasing symbiont exploitation of the host. Further, we find the surprising result that vertical transmission can inhibit benefit evolution by interfering with the positive influence of horizontal transmission. These findings apply to a wide range of systems and can be extended to the evolution of horizontally transferred genes, such as those underlying antibiotic resistance, as well as to eukaryotic interactions such as between figs and fig wasps.

## The Modeling Approach

All models employed are based on a general backbone that describes the ecological dynamics of horizontally transmitted symbionts regardless of their effects on host fitness. We first describe this general form before expanding to include the details of different mechanisms that link horizontal transmission and symbiont-provided benefits.

### Ecological dynamics

We consider interactions between hosts and symbionts, in which symbionts replicate within hosts and may survive (but not reproduce) in the external environment. Host population dynamics are assumed to follow logistic growth. For simplicity, we assume that multiple symbionts cannot infect the same host. Extension of the models to incorporate within-host symbiont competition goes beyond the scope of this work but may be the focus of a future study. We use “infect” to connote a life-cycle stage that initiates the interaction between the host and symbiont, rather than implying that the symbiont is a parasite.

We start with a susceptible-infected model that includes the effects of uninfected immigration and imperfect vertical transmission of the symbiont. Immigration of susceptible hosts and imperfect vertical transmission provide two levers for manipulating transmission mode in the model without directly altering the traits at the focus of the study (i.e., symbiont-provided benefits and transmission rate). The model backbone is given by:


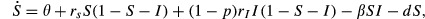
(1a)


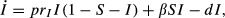
(1b)

where *S* and *I* are the densities of susceptible and infected hosts, *θ* is the uninfected immigration rate, *p* is the rate of vertical transmission, *β* is the overall rate of horizontal transmission, *r*_*S*_ and *r*_*I*_ are the growth rates of susceptible and infected hosts, and *d* is the rate of extrinsic mortality. Note that this formulation assumes symbionts only affect host reproduction (or more specifically, the net growth rate), whereas models of parasite virulence evolution often assume parasites affect host survival (e.g., Lipsitch et al. 1996). We will focus on effects of symbionts on host reproduction, as this captures the biology in the specific scenarios modeled below. Additionally, the term *βSI* in the model suggests direct transmission of symbionts between hosts, although the same model can also approximate environmental acquisition of symbionts by decomposing *β* into terms for symbiont fecundity and the efficiency of infection. Table1 summarizes all variables, parameters, and parameter values used in the models.

**Table 1:** Summary of notation used in each scenario.

Symbol	Description (value used in analysis, where applicable)	Scenario
*S*	Susceptible host density	All
*I*	Infected host density	All
*r*_*S*_	Intrinsic rate of (uninfected) host growth (1, unless a function)	All
*r*_*I*_	Intrinsic rate of infected host growth	–
*β*	Intrinsic rate of infection (10^−4^, unless a function)	All
*d*	Extrinsic mortality rate (10^−5^)	All
*a*	Probability of successful infection after contact (1, unless a function)	All
*f*	Symbiont fecundity (1, unless a function)	All
*b*	Symbiont-encoded benefits	All
*θ*	Migration rate (varies as described in figures)	All
*p*	Probability of successful vertical transmission	All
S^*^	Equilibrium density of susceptible hosts	All
I^*^	Equilibrium density of infected hosts	All
*λ*	Invasion fitness of a mutant symbiont	All
*c*	Costs of infection (0.4 in partner choice model)	All
*z*	A generic trait underlying one or more model parameters	Pleiotropy
*γ*	Baseline symbiont fecundity for partner choice (5)	Partner choice
*ε*	Trade-off between fecundity and benefits for partner choice (2)	Partner choice
*τ*	Maximum infection rate (0.01)	Partner choice
*ϕ*	Choice threshold (varies as described in figures)	Partner choice
*c*_*h*_	Host waste production and associated cost	By-products
*q*	Proportion of host waste consumed by symbiont	By-products
*σ*	Describes relationship between host waste production and host growth (1.25)	By-products
*δ*	Marginal cost of symbiont fecundity to host (0.50)	By-products
*c*_*s*_	Total cost due to symbiont fecundity	By-products

Model (1) has three potentially stable equilibria, one where all individuals are susceptible (“All-S”), one where all individuals are infected (“All-I”), and one where susceptible and infected individuals coexist (“Co-X”; see Appendix A1 for solutions). Although the equilibria are analytically solvable, their stability analysis is not analytically tractable. Numerical explorations, however, show that Co-X prevails through most of the parameter space for both parasites (symbionts reduce host growth rate: *r*_*I*_ < *r*_*S*_) and mutualists (symbionts increase host growth rate: *r*_*I*_ > *r*_*S*_), with All-S only occurring when the overall rate of horizontal transmission, *β*, is lower than the death rate, and vertical transmission, *p*, is low relative to the migration rate, *θ* (see Supporting Information). Throughout this work, we avoid migration rates in excess of the death rate, because such a parameter space would force the total population density to exceed the carrying capacity, giving rise to a “pseudo-sink” in which the individuals with higher growth rates (e.g., mutualist-infected hosts) would have lower fitness as a result of stronger competition (Watkinson and Sutherland 1995). Although such effects may arise under certain natural conditions, their inclusion in the parameter space would confound the effects of trait correlations at the focus of this study. For the remainder of this study, we restrict our attention to a broad parameter space in which susceptibles and infecteds coexist, as this equilibrium includes both moderate parasites and all mutualists and is the only equilibrium under which we can explore symbiont evolution.

### Evolutionary dynamics

Given model (1), we then follow an adaptive dynamics approach for predicting trait evolution under several natural scenarios. Adaptive dynamics is a form of continuous game theory that uses stability analysis to determine the ability of a rare mutant to invade a resident population (Dieckmann 1997; Waxman and Gavrilets 2005; Otto and Day 2007). This method assumes that the resident population is at a stable ecological equilibrium (here, Co-X), that trait values map continuously onto fitness and that individual mutations are of relatively small effect (Geritz et al. 1998; Otto and Day 2007). Adaptive dynamics (in a variety of forms) has been widely used in previous models of parasitism and mutualism evolution (e.g., Yamamura 1996; van Baalen 1998; Ferriere et al. 2002; Holland et al. 2004; Restif and Koella 2004).

For all scenarios explored here, we assume the population is at demographic equilibrium and consider under what conditions mutant symbionts will have a positive relative growth rate and will, therefore, invade the resident symbiont population. The growth of hosts infected by mutant symbionts is given by the following:


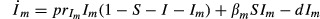
(3)

where subscript *m* indicates that the variable may take a mutant value. Equation (2) provides the growth rate of hosts infected by mutant symbionts as a function of the equilibrium densities of the resident susceptible and infected host subpopulations. We can approximate the invasion fitness, *λ*, of a mutant symbiont by differentiating (2) with respect to *I*_*m*_ and evaluating the result when *I*_*m*_ is zero. When the invasion fitness is positive, a single mutant can invade the resident population. Evolutionary stable strategies (ESS) are trait values that cannot be invaded by a mutant, and they are determined for each trait *z* by solving 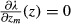 and confirming that the result is a local maximum (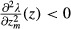). Mutant invasion fitness, *λ*, will generally be of the form:


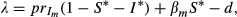
(4)where S* and I* are densities at demographic equilibrium.

### A general outcome when 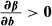


We first examine the general effects of a positive relationship between transmission rate and symbiont-provided benefits on symbiont invasion fitness using equation (3). We will suppose that *r*_*I*_ = *r*_*S*_ + *b* – *c*, where *b* and *c* are the benefits and costs conferred by the symbiont to the infected host. We will generally be concerned with how two model parameters affect the relationship between horizontal transmission and benefit evolution: the migration rate, *θ*, which modulates the availability of susceptible hosts, and the vertical transmission rate, *p*, which determines what portion of an infected host's offspring will be infected. In order to solve for the evolutionary stable level of benefits provided by symbionts to hosts, b^*^, we substitute this expression for *r*_*I*_ into (3) and differentiate with respect to *b*_*m*_:


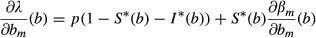
(5)

From (4), we can determine when symbiont invasion fitness will increase as a result of providing the host with greater benefits. In general, the total host density at equilibrium will be below the carrying capacity (i.e., S* + I* < 1), unless the migration rate is sufficiently greater than the death rate, *d*, to artificially maintain a large population size. For any mechanism that creates a positive relationship between symbiont horizontal transmission and the benefits provided by symbionts to hosts (i.e., 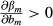 ), benefits will increase indefinitely unless limited to a maximum level determined by other genetic or physiological constraints. In reality, the sign of 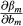 may vary based on the mechanistic details of the system in question and the resident value of *b*.

Within this context, pleiotropy is the simplest mechanism that can create a positive covariance between symbiont-provided benefits and horizontal transmission rate. One possibility would be that the traits underlying both symbiont-provided benefits and horizontal transmission are affected by expression of the same gene(s) (or may even be different functions of the same gene), as may be the case for luminescence and motility in *V. fischeri* (Visick et al. 2000) or for conjugation rate and tetracycline resistance in the plasmid pB15 (Turner et al. in press[Bibr b47]). For this scenario, we can then ask how changing the rates of migration or vertical transmission would affect the evolution of benefits. In what follows, it is useful to keep the example of pleiotropy in mind, although it should be noted that any genetic mechanism that creates a positive covariance between horizontal transmission and symbiont-provided benefits should have the same effect on evolution. To do so, we differentiate (4) with respect to *θ* or *p* and find:


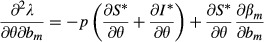
(6)


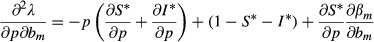
(7)

**Figure 1:**
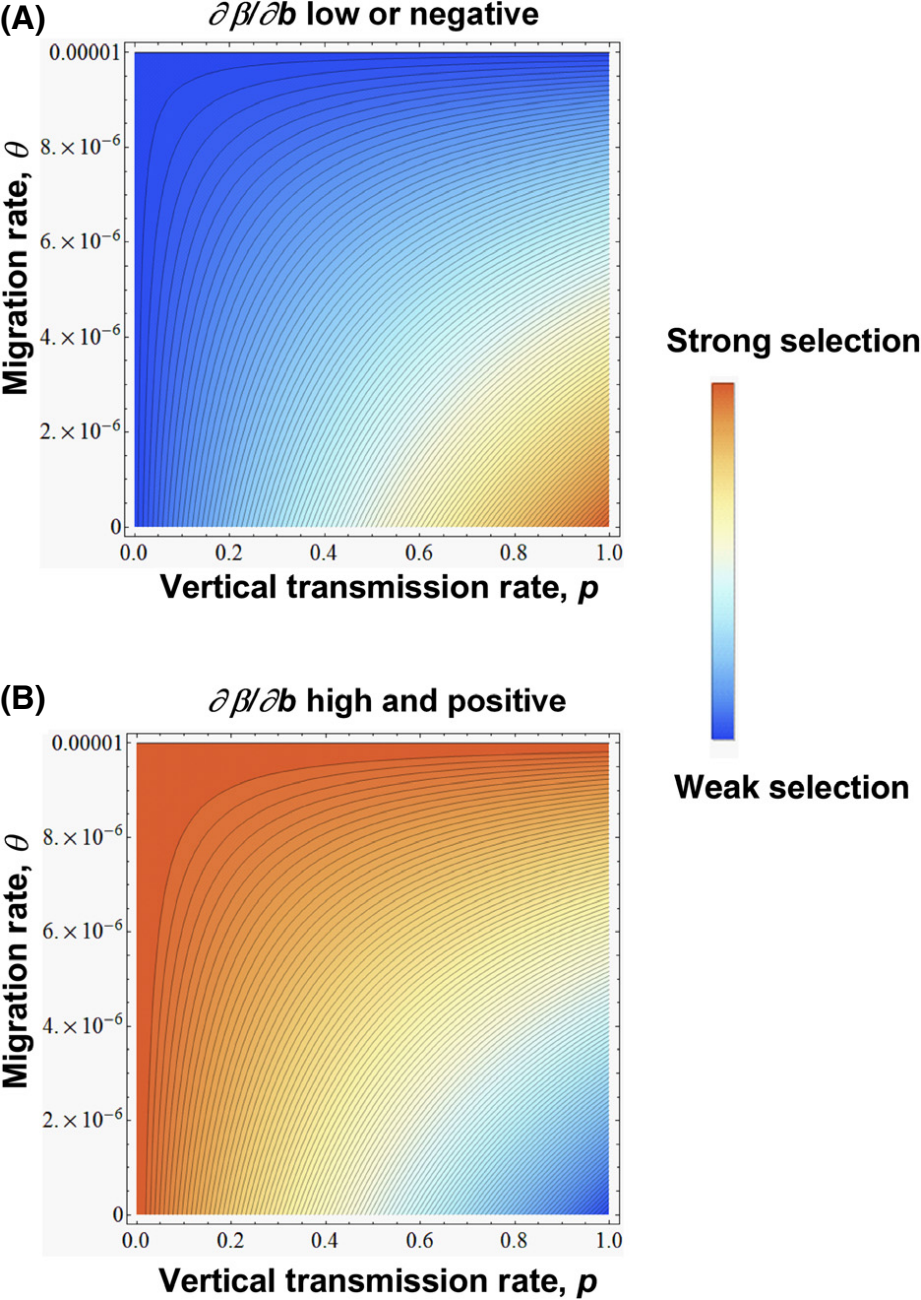
Contour plots for the strength of selection on benefits as a function of vertical transmission, *p*, and migration rate, *θ*, when 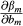 is negative or only weakly positive (A: 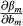 = 5 × 10^−6^) or when sufficiently large and positive (B: 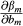 = 5 × 10^−4^). Other parameters used: *r*_*I*_ = 1.25, *r*_*S*_ = 1.00, *d =* 1 × 10^−5^, *β* = 1 × 10^−4^.

We examine the effects of *θ* first. In general, increasing *θ* will increase the equilibrium density of susceptibles, S*, and decrease the equilibrium density of infecteds, I*, and will cause a slight increase in the total equilibrium host population. As a result, the first term in (5) will always be negative (see Supporting Information). Further, because 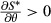 and we assume 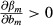, the second term in (5) will always be positive. In order for (5) to be positive, 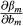 must be large enough to overcome the selective interference created by vertical transmission through the first term in the equation (Fig.1). Interestingly, this also implies that as the rate of vertical transmission, *p*, approaches zero, the impact of horizontal transmission on benefit evolution will increase. In brief, when there is a sufficiently strong, positive correlation between horizontal transmission rate and symbiont-provided benefits, increasing the rate of migration will generally amplify any indirect selection to increase benefits resulting from direct selection to increase horizontal transmission.

Vertical transmission has the opposite effect from migration on susceptible host density, and increasing *p* will reduce S*. Further, numerical exploration of the first term in (6) demonstrates that the net effect of *p* on total host density, S* + I*, is essentially negligible (see Supporting Information). As a result, (6) will only be positive when *θ* and 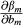 are low enough to ensure 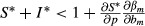. In other words, increasing *p* will increase the effect of benefits on mutant symbiont invasion fitness whenever there is at most a weak positive relationship between horizontal transmission and symbiont-provided benefits. If, instead, there is a strong positive correlation between these traits and this inequality fails to hold, we again find that increasing the rate of vertical transmission will inhibit the evolution of higher benefits (Fig.1). Note that this can occur if migration, *θ*, is high enough to force a high total host density (i.e., S^*^ + I^*^ > 1), or if 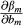 is sufficiently high. In the former case, vertical transmission reduces selection on benefits, because migration pushes the population beyond carrying capacity. As noted above, this scenario describes a pseudo-sink and we will generally avoid this part of parameter space. In the latter case, vertical transmission interferes with the positive effects of horizontal transmission by reducing the number of susceptible hosts.

Overall, we find two notable outcomes when symbiont-provided benefits positively covary with symbiont horizontal transmission: (1) horizontal transmission may increase selection acting on symbionts to benefit their hosts, and (2) vertical transmission can interfere with this selection by reducing the number of susceptible hosts. Further, while pleiotropy is the simplest mechanism that supports these predictions, the results should hold for any mechanism that establishes a positive relationship between the rate of infection and the benefits that symbionts provide their hosts.

We next extend these results to cases when 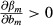 as a result of either partner choice by the host or of symbiont consumption of host waste by-products.

### Partner choice

Mechanisms of partner choice include both favoring more beneficial symbionts (e.g., Zhang et al. 2007) and punishing less beneficial or more costly partners (often called sanctions; e.g., Kiers et al. 2003). In each case, partner choice establishes a positive covariance between horizontal transmission and the net benefits provided by symbionts to hosts. Our focus here is how this effect of partner choice on symbiont trait covariances will affect the evolution of symbiont-provided benefits. To do so, we consider an example of partner choice in which hosts favor infection by more beneficial (and/or less costly) symbionts. As a result, the host phenotype can cause 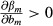.

To incorporate host choosiness into the model, we first let *β* = *af*, where *a* is the probability that contact between an uninfected host and a free symbiont results in infection, and *f* is the number of free symbionts produced per infected host (symbiont fecundity). Host choosiness is then included by allowing *a* to be a sigmoidal function of the benefit/cost ratio, *b/c*, given by:


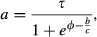
(8)

where *τ* is the maximum infection rate, and *ϕ* determines the threshold value of *b/c* necessary to obtain a particular transmission rate, with higher *ϕ* corresponding to higher thresholds. Similar functions have been used to describe mate choice decisions as a function of a partner's expression of a desirable phenotype (Kazancioglu and Alonzo 2012). Note that the choice to model the rate of infection as a function of *b/c* rather than (*b – c*) is one of mathematical convenience, and similar results would be obtained by rescaling the other parameters in (7).

In nature, partner choice may be especially important whenever providing benefits is costly to symbionts. We incorporate these costs by assuming that the number of symbionts produced by an infected host, *f*, trades off with symbiont-provided benefits, *b,* according to the linear function:


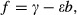
(9)where *γ* is the maximum rate of free symbiont production, and *ε* determines the marginal cost of increased benefit production.

Note that equations (7) and (8) are at odds with one another over how providing benefits will affect symbiont horizontal transmission. Given these functions, we follow standard adaptive dynamics to determine how changing the rates of migration or vertical transmission will affect the ESS level of benefits provided by symbionts to their hosts.

**Figure 2:**
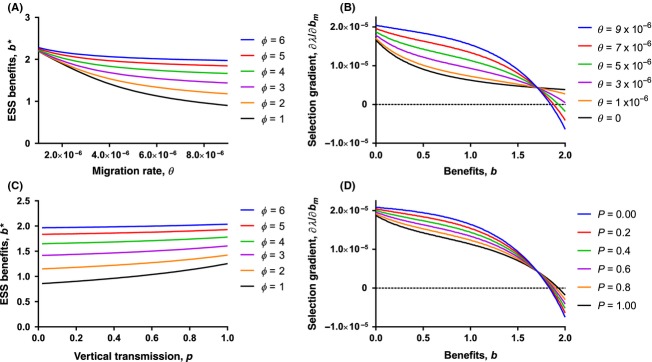
(A) Each line describes the Evolutionary stable strategies (ESS) benefit level for a given migration rate, *θ*, and partner choice strength, *ϕ*. (B) Each line shows the selection gradient, 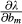, as a function of the current benefit level, *b*, for migration rates ranging from *θ* = 0 to *θ =* 9 × 10^−6^ (and shown for *ϕ* = 5, *p =* 1). (C) Each line describes the ESS benefit level for a given vertical transmission rate, *p*, and partner choice strength, *ϕ*. (D) Each line shows the selection gradient, 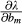, as a function of the current benefit level, *b* for vertical transmission ranging from *p* = 0 to *p* = 1 (and shown for *ϕ* = 5, *θ* = 5 × 10^−6^).

We first examine the effect of migration rate on the ESS level of benefits given the strength of host choice, *ϕ*. As shown in Figure2A, as hosts become choosier (*ϕ* increases), the ESS benefit level increases for all levels of migration, *θ*. Further, for a given *ϕ*, ESS benefits decrease as the migration rate increases. This would suggest that increasing the migration rate has an obligate, negative effect on benefit evolution. As Figure2B shows, this result does not tell the entire story. If, instead, we examine the strength of selection on benefits, as measured by 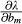, we find that increasing *θ* generally increases the strength of selection on benefits provided by the symbiont to the host, as expected from our earlier analysis of the general problem. Further, it is reasonable to assume that benefit production may be limited to a maximum value in natural systems. If this is the case and *ϕ* is sufficiently high, then symbionts will evolve the maximum level of benefit, regardless of the migration rate. In fact, higher *θ* will increase the strength of selection, meaning that greater horizontal transmission may lead to more rapid evolution of maximum benefits when partner choice is strong.

The rate of vertical transmission, *p*, also plays an important role in shaping the effects of partner choice and interacts strongly with the rate of migration. As seen in Figure2C, increasing the rate of vertical transmission will favor greater benefits. The marginal effect of increasing vertical transmission, however, declines as the rate of migration increases toward the death rate. Further, higher vertical transmission also leads to weaker selection on benefits (Fig.2D). The reasoning for this effect is similar to that explored in the previous section. When *θ* is low, vertical transmission has a stronger relative effect than horizontal transmission on symbiont fitness, and increasing *p* amplifies this effect. Inversely, when *θ* is high, horizontal transmission has a greater relative impact on symbiont fitness, and increasing *p* will actually dampen that positive effect on benefit evolution. Moreover, if symbionts are physically limited to provide a benefit level below the ESS, greater vertical transmission may not actually lead to symbionts that provide higher benefits, but only to less rapid evolution of those benefits.

### By-products

We next consider a by-product interaction in which host reproduction results in within-host accumulation of a waste by-product, at rate *c*_*h*_, of which symbionts consume a portion, *q*. Greater host growth entails greater production of waste, but high waste production will ultimately inhibit further host growth. Symbionts, therefore, indirectly benefit hosts by reducing the costs of greater host reproduction. Further, symbiont fecundity rises with increased consumption of host waste but at a diminishing rate. We also assume that symbiont reproduction is itself costly to the host at a rate, *c*_*s*_, proportional to symbiont fecundity. We again decompose *r*_*I*_ into *r*_*S*_ *+ b – c* and *β* into *af*, where *a* is now held constant. *c* is taken to be the sum of the costs to the host due to its own waste production, *c*_*h*_*,* and due to symbiont reproduction, *c*_*S*_. The following functional forms define the remaining variables:


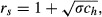
(10)


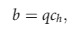
(11)


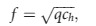
(12)


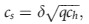
(13)where parameters are as described above, and where *σ* and *δ* are scaling parameters. As noted previously, host growth is assumed to increase in *c*_*h*_ to a point and then decline, whereas the costs and benefits of infection by the symbiont are both increasing functions of *c*_*h*_ and *q*. Parameter choices throughout the model were chosen to constrain the population dynamics to the same co-existence equilibrium as in the other scenarios.

We then follow the same approach as for pleiotropy and partner choice to determine the evolutionarily stable portion, q*, of host waste consumed by symbionts. Although it would also be of interest to solve for optimal host waste production, the formulation of immigration in the model makes analyzing host coevolution intractable without assuming that immigrant traits perfectly track the evolving host population. We instead maintain our focus on the effects of immigration rate and vertical transmission on the evolution of the symbiont trait, *q*. Future work requiring a different immigration model may investigate the important effects of host coevolution.

**Figure 3:**
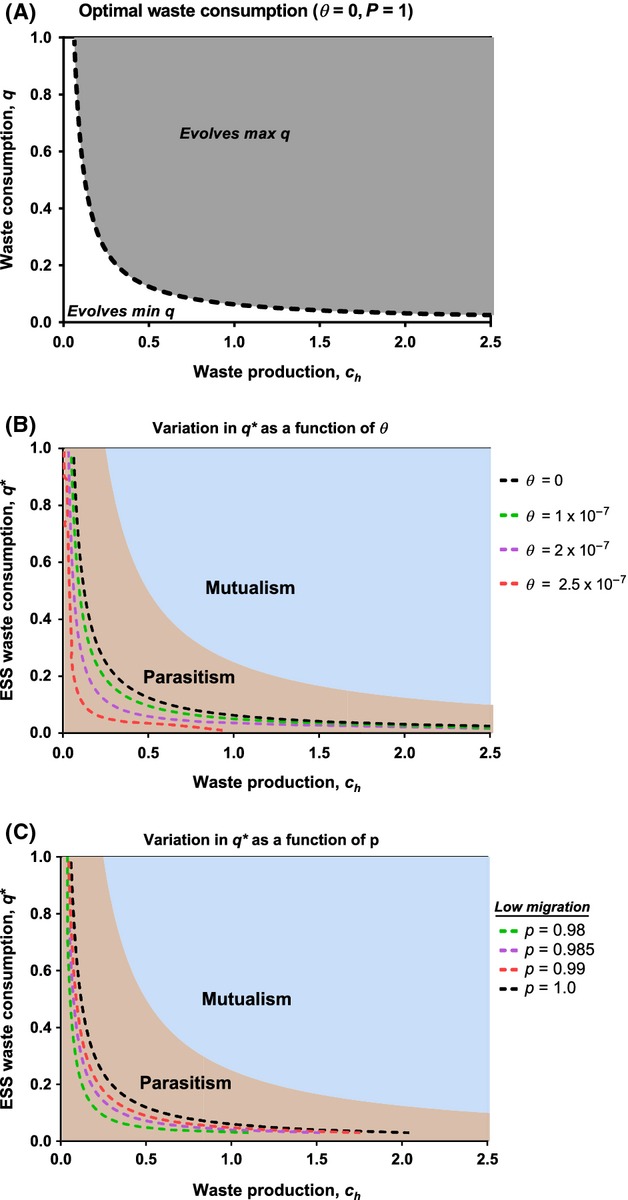
(A) Evolution with only vertical transmission (*θ* = 0, *p* = 1). The dashed line defines the unstable equilibrium level, q*, of waste consumption by symbionts as a function of host waste production, *c*_*h*_. When initial trait values are to the right (left), symbionts evolve maximum (minimum) *q*. (B) Increasing *θ* shifts this threshold toward the origin (*θ* = 0, 1 × 10^−7^, 2 × 10^−7^, 2.5 × 10^−7^). (C) In contrast, increasing *p* shifts the unstable boundary away from the origin (*p* = 0.98, 0.985, 0.99, 1.00). The mutualistic trait space is shaded blue, and the parasitic space is shaded orange.

In general, the equilibrium exploitation of host waste by symbionts is a decreasing function of host waste production (Fig.3A,B). Further, this equilibrium is always unstable, unless we relax the assumption that migration rate is lower than the death rate (forcing the population dynamics into a pseudo-sink). As a result, symbionts will evolve either minimum or maximum exploitation of host wastes, depending on the initial trait values (Fig.3A). The former corresponds to a parasitic outcome, whereas the latter leads to the evolution of mutualism. As *θ* increases toward the death rate, this boundary shifts toward the origin, resulting in a larger portion of initial trait values that support the evolution of mutualism (Fig.3B). Thus, rare immigration events may enable the evolution of mutualism under conditions that would otherwise favor parasitism evolution. Further, reducing the rate of vertical transmission also shifts this unstable equilibrium toward the origin, indicating that lower rates of vertical transmission will also support the evolution of mutualism for a larger set of initial trait values (Fig.3C). Taken together, these predictions recapitulate the general results found for pleiotropy and for partner choice.

## Discussion

In this study, we set out to understand how horizontal and vertical transmission affect the evolution of benefits provided by symbionts to hosts. We explored this question in a general sense, taking pleiotropy as a basic underlying mechanism, and then in more detail in examples of partner choice and by-product interactions. Across all scenarios, we found conditions under which horizontal transmission is expected to increase selection on symbiont-provided benefits. We also found that increased rates of vertical transmission will often reduce the strength of selection on these benefits.

### Model assumptions and future extensions

As in all mathematical models, this study relies on several assumptions in order to preserve analytical tractability. In the present work, these assumptions also help to maintain focus on the effects of horizontal and vertical transmission. That said, a number of these assumptions may be worth relaxing and exploring in greater detail in future studies.

In particular, we considered population dynamics for symbionts that do not experience interspecific or intraspecific competition with other symbionts within individual hosts. Within-host competition can significantly alter the evolution of virulence in parasites (Bull 1994), and within-host competition might be expected to dampen the effects of a positive covariance between horizontal transmission and symbiont-provided benefits. At the same time, several other model assumptions would need to be considered to account for possible covariances between the success of symbionts competing within hosts and the benefits received by hosts. For instance, in the case of by-product mutualisms in which symbiont exploitation of host wastes confers an indirect benefit to hosts, within-host competition may be expected to increase the total exploitation of host waste by all symbionts within a single host, which may, in fact, promote rather than hinder the evolution of mutualism.

Further, we have examined the effects of pleiotropy, partner choice, and by-products in isolation, but some combination of these processes may be at work in natural systems, and the nature of the partner choice or by-product exchange may vary. In the case of *V. fischeri* and bobtail squid, pleiotropy and partner choice may both take place (Visick and McFall-Ngai 2000; Visick et al. 2000); in the interaction between legumes and rhizobia, the mutualism hinges on the reciprocal exchange of by-products (rather than waste removal alone), and hosts allocate fewer resources to nodules harboring less helpful symbionts (Kiers et al. 2003). We have also focused on the effects of symbionts on host reproduction, although it is possible that some results might change if considering effects of symbionts on host survival. It is not immediately obvious, though, how these distinctions might affect the interaction between transmission mode and the evolution of symbiont-provided benefits.

Finally, we have restricted our attention entirely to symbiont evolution, although host coevolution must play an important role in nature. In a general sense, it would be interesting to explore how host coevolution of resistance or tolerance strategies affects the evolution of symbiont-provided benefits. The notion that host tolerance evolution may promote the ultimate evolution of mutualistic symbionts has gained some attention in recent years (Edwards 2009; Oliver et al. 2009), and such predictions would likely be compatible with the results discussed in the present study. In the more specific case of partner choice, the null expectation is that host coevolution should favor the evolution of choosier hosts. And in by-product interactions, hosts may reduce waste production (and therefore their own growth potential) if symbionts are not sufficiently exploitative but may increase waste production when symbionts are capable of removing large portions of that waste. In either case, it is unclear if host coevolution would qualitatively alter the predicted effects of immigration rate and vertical transmission.

### The conflict between partner choice and vertical transmission

While the models in this study often assume that both vertical and horizontal transmission will be possible for the symbiont, many natural examples lack vertical transmission entirely, such as in the case of the gut symbionts of stinkbugs (Kikuchi et al. 2007). Although we have not modeled the evolution of vertical transmission rate, the results imply the potential for conflict between the partner fidelity feedback created by vertical transmission and the mechanisms underlying a positive covariance between horizontal transmission and symbiont-provided benefits. In the case of partner choice, in particular, we are unaware of any natural examples that also rely on direct vertical transmission. This may be because partner choice itself requires horizontal transmission for hosts to choose between symbionts (Usher et al. 2007) and thereby creates an inherent conflict between vertical transmission and partner choice. Our results imply that this conflict may also be due to the effects of vertical transmission on population dynamics and the resultant reduction in selection on symbiont-provided benefits.

### Implications for disease evolution and nonmicrobial systems

Although we have largely focused our attention on examples of mutualistic symbionts, the results also have implications for bacterial pathogens that benefit from their own symbionts in the form of conjugative plasmids and bacteriophages. Conjugative plasmids, for instance, often carry genes for antibiotic resistance, and it has been demonstrated that there can be a strong positive correlation between the level of antibiotic resistance and the rate of plasmid transfer between bacteria (Turner et al. 1998, in press[Bibr b47]). Additionally, some gram-positive bacteria, such as *Enterococcus faecalis*, rely on a pheromone system of plasmid transfer (Clewell 2007). In these opportunistic pathogens, plasmid-free bacteria use a chemical signal to upregulate conjugation with plasmid-bearing bacteria in environments containing antibiotics (Clewell et al. 2002). As such, resistance in *E. faecalis* may evolve according to a partner choice process and may also provide an excellent model system for testing the partner choice results discussed above.

Bacteriophages (phage), and filamentous phages in particular, have also been implicated in the pathogenicity of a wide variety of agricultural pests and human pathogens, ranging from wilt disease to cholera (Waldor and Mekalanos 1996; Addy et al. 2012). The present study suggests that it is worth exploring possible correlations between phage traits associated with both phage transmission and phage-encoded virulence factors. If these traits can be positively correlated, then increased horizontal transmission of these viruses between their bacterial hosts may significantly affect the evolution of virulence of the infected bacteria toward their own eukaryotic hosts.

Many of the predictions reported here may also extend beyond microbial symbionts. In the case of fig wasps, wasps pollinate within figs while laying their eggs. In some instances, if a wasp lays eggs without pollinating, the fig is aborted, killing the unborn wasps (i.e., partner choice; Jander and Herre 2010). Mathematically, little distinguishes the fig wasp and other such pollinators from a microbial symbiont, and we expect the predictions in this study to hold in such cases, as well as in any others in which the model backbone would not be significantly perturbed by biological idiosyncrasies.

Overall, this study provides several new hypotheses for how transmission mode may affect the evolution of benefits that symbionts provide hosts. Not only can increasing susceptible host availability result in increased selection for greater symbiont-provided benefits, but vertical transmission may interfere with this selection. These predictions are in contrast to typical expectations in the mutualism and parasitism literature and offer a new perspective for future models and experiments.

## Appendix A1: Ecological equilibria

There are four possible solutions to model (1), two of which are excluded as they give negative densities. The remaining equilibria are as follows:

### All-Susceptible (All-S)

(A1)

### Coexistence (Co-X)

To improve the readability of the following equations, we substitute:

We then find:

(A2a)

(A2b)

Note: The Co-X equilibrium becomes an All-Infected (All-I) equilibrium when there is perfect vertical transmission (*p* = 1) and no immigration (*θ* = 0).

Stability analysis was performed numerically and can be found in the Supporting Information.

## Appendix A2: Invasion fitness derivations

As noted in the main text, the ESS value of a trait *z* is found by solving for when  and , given the conditions of each scenario. In the case of partner choice, we solved for the symbiont's ESS benefit level, *b*^***^. To do so, we substituted equilibrium (A2) and trait functions (7) and (8) into (4) to derive  and . We then used *Mathematica*'s FindRoot function to numerically solve for the roots of . Finally, we confirmed that each solution was an ESS by substituting the solution for *b*^***^ into .

For by-product interactions, we used similar techniques to solve for the equilibrium portion of host waste consumed by the symbiont, *q**, again using equilibrium (A2) and now trait functions (9–12). Solving for the second derivative, , indicated that this equilibrium is unstable ( ) when the migration rate, *θ* is below the death rate, *d*.

## Supporting Information

**Data S1.** Stability analysis. **Figure S1**. Each graph shows the region supporting the Co-X (purple) and All-S (tan) equilibria, across values of migration rate, *θ* and vertical transmission rate, *p*. **Figure S2**. Host density at equilibrium across values of *p* and *θ* for susceptibles (a), infecteds (b) and the total host population (c).
