# Supplementary material for: The impact of transmission mode on the evolution of benefits provided by microbial symbionts
Source: Ecol Evol. 2014 Aug 6;4(17):3350–61. doi: 10.1002/ece3.1166 (PMC4228610; doi:10.1002/ece3.1166)
Supplement: Supplementary file 1 [file ece30004-3350-sd1.docx]

**Supporting Information: Stability analysis**

In this study, we employed the following SI model:

$\dot{S}=\theta+r_{S}S\left( 1-S-I \right)+\left( 1-p \right)r_{I}I(1-S-I)-\beta SI-dS$ (S1a)

$\dot{I}=pr_{I}I\left( 1-S-I \right)+\beta SI-dI$ (S1b)

As noted in the main text, while the Jacobian matrix for (S1) and its corresponding eigenvalues are solvable under either the All-S or Co-X equilibrium, interpretation of the eigenvalues for the purposes of stability analysis is not readily done. To this end, we used *Mathematica* 9.0 to numerically solve for the eigenvalues under each of the possible equilibria given a range of parameter values. We then used these solutions to construct phase diagrams showing the regions of parameter space that support each of the equilibria (Fig. S1). Figure S1 shows that decreasing the rate of infection increases the range of parameter values that support the All-S equilibrium. When the rate of infection is greater than the death rate, the Co-X equilibrium will always be stable. If, however, the infection rate is lower than the death rate, then a portion of parameter space will support the All-S equilibrium. Given such parameters, a Co-X equilibrium can shift toward the All-S equilibrium by either increasing the rate of migration (more susceptibles forced into the system) or decreasing the rate of vertical transmission (fewer infected individuals maintained). Additionally, for very low infection rates, the majority of parameter space may favor All-S. In the main text, we generally restrict our attention to infection rates high enough such that the entire range of migration and vertical transmission rates used support the Co-X equilibrium. Note also that while Figure S1 only shows results when symbionts are mutualists (*r_I_ > r_S_*), similar predictions hold for moderately harmful parasites (not shown).

*Effect of changing* p *and* θ *on total host density at equilibrium*

Figure S2 shows how changing *p* and *θ* affect the density of susceptibles, infecteds, and the total host population. Increasing *θ* will increase the number of susceptibles, decrease the number of infecteds, and slightly increase the total host population. Increasing *p* has a weak, negative effect on susceptibles, a slightly stronger, positive effect on infecteds, and a very weak positive effect on the total host density that is essentially negligible except for when migration is very low.

**Figure S1**. Each graph shows the region supporting the Co-X (purple) and All-S (tan) equilibria, across values of migration rate, *θ* and vertical transmission rate, *p*. (a) *β =* 10^-5^ = *d* (same results if *β* > *d*), (b) *β* = 9 x 10^-6^, (c) *β =* 7 x 10^-6^, (d) *β* = 1 x 10^-6^. Other parameters used: *r_I_* = 1.25, *r_s_* = 1.00, *d* = 10^-5^.

(a) *β =* 10^-5^


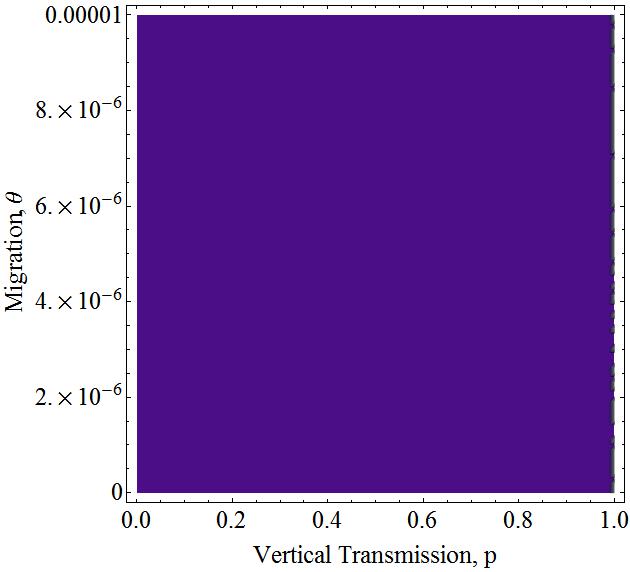


Co-X

(b) *β =* 9 x 10^-6^


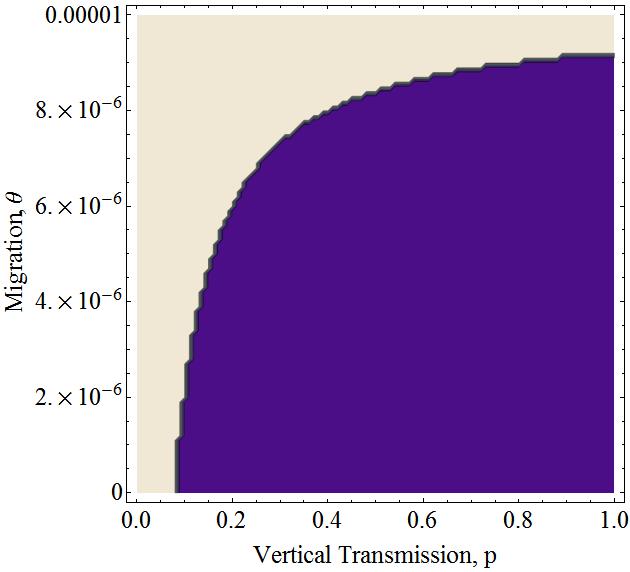


All-S

Co-X

(c) *β =* 7 x 10^-6^


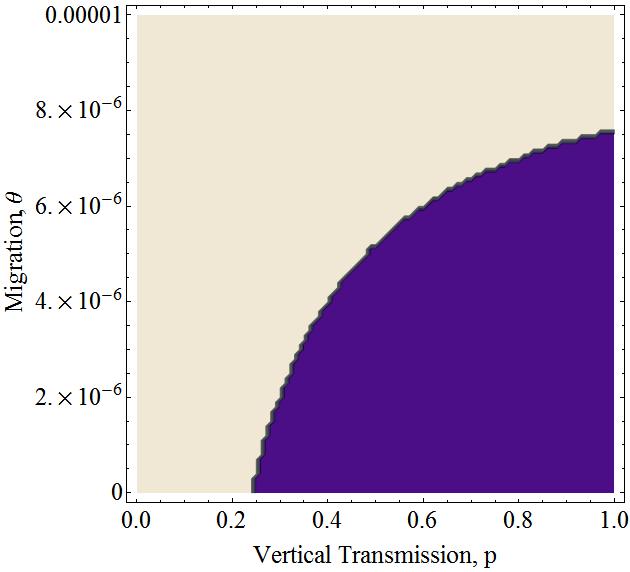


All-S

Co-X

(d) *β =* 10^-6^


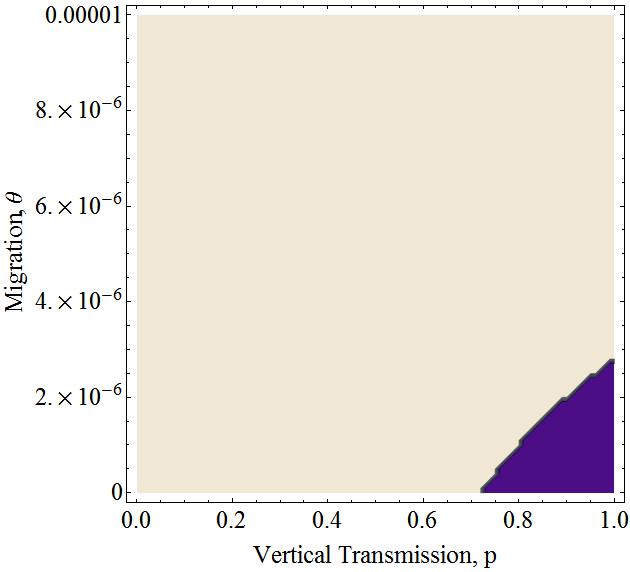


All-S

Co-X

**Figure S2**. Host density at equilibrium across values of *p* and *θ* for susceptibles (a), infecteds (b) and the total host population (c). Parameters are the same as in Figure S1a (*r_I_* = 1.25, *r_s_* = 1.00, *d* = 10^-5^, *β* = 10^-5^).

(a) Effect of parameters on Susceptibles

**
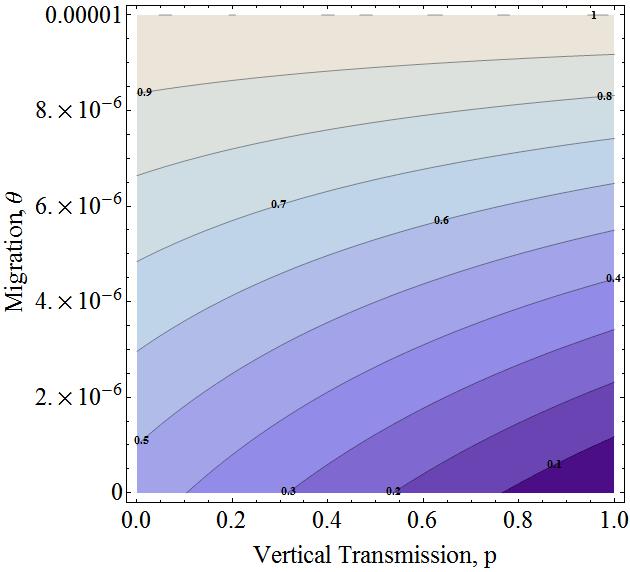
**

(b) Effect of parameters on Infecteds


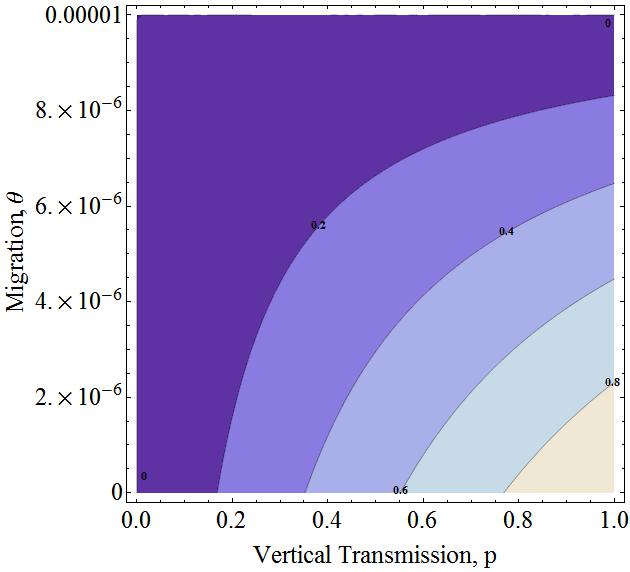


(c) Effect of parameters on total host density

**
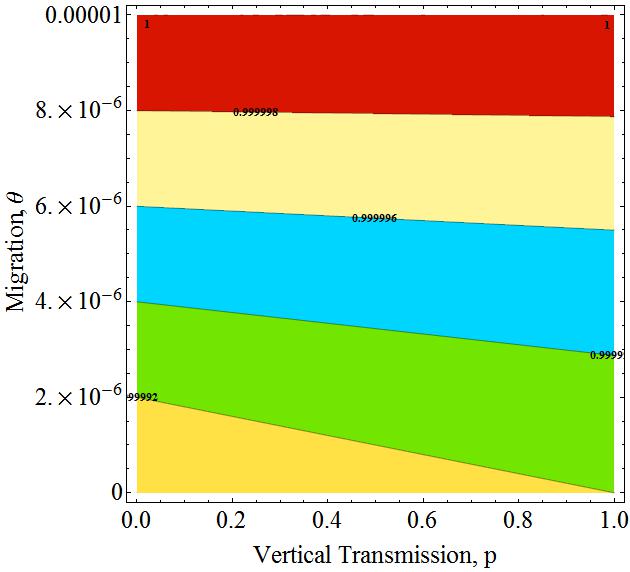
**
